# Ultrasound-Guided Dorsolateral Approach for Quadratus Lumborum Block in Rabbits (*Oryctolagus cuniculus*): A Prospective, Randomized, Blinded, Cadaveric Study Comparing Four Different Injectate Volumes

**DOI:** 10.3390/ani13162559

**Published:** 2023-08-08

**Authors:** Lucía Torres Cantó, Ricardo Felisberto, Anita Economou, Derek Flaherty, Beatriz Moreno Aguado, Hamaseh Tayari

**Affiliations:** Southern Counties Veterinary Specialists, Forest Corner Farm, Hangersley, Ringwood BH24 3JW, UK; luciatorrescanto@gmail.com (L.T.C.); ricardo.felisberto@scvetspecialists.co.uk (R.F.); anita_economou@yahoo.com (A.E.); derek.flaherty@scvetspecialists.co.uk (D.F.); beatriz.moreno@scvetspecialists.co.uk (B.M.A.)

**Keywords:** loco-regional anesthesia, analgesia, rabbit, ultrasound-guided, quadratus lumborum block, fascia, interfascial plane block

## Abstract

**Simple Summary:**

The popularity of rabbits as pets has grown substantially in the UK. However, despite progress, this species still has a much higher anesthesia-related mortality than dogs and cats. Rabbits are prey animals that tend to hide signs of pain, which makes it particularly difficult to assess and to treat it adequately. Perioperative systemic opioid administration is a cofactor in the high perioperative mortality and morbidity rates recorded in rabbits. To reduce post-surgical opioid requirements, a multimodal analgesia regimen is advisable. In such a context, loco-regional anesthesia has gained a central role in daily anesthesia practice as it effectively and significantly reduces the doses of perioperative systemic analgesic drug requirements. The quadratus lumborum block (QLB) is an ultrasound-guided loco-regional anesthesia technique developed in human medicine consisting of the injection of local anesthetic within the quadratus lumborum (QL) fascia that provides analgesia to the abdominal wall and viscera by blocking the ventral branches of the thoraco-lumbar spinal nerves and sympathetic trunk. The efficacy of the QLB in human medicine has driven veterinary research to investigate this block in companion and farm animals. To date, no study has investigated the QLB in rabbits. With the present cadaveric study, these authors aimed to carry out the following: firstly, to investigate the anatomical layout of the thoraco-lumbar area in rabbits; secondly, to design a feasible ultrasound-guided QLB for rabbits; and thirdly, after comparing different volumes of injectate and analyzing the spread obtained, to propose the most suitable volume of injectate to administer. This research was conducted by performing anatomical dissections and computed tomographic studies in rabbit cadavers.

**Abstract:**

Ultrasound-guided (US-guided) loco-regional anesthesia can provide significant analgesia and anesthetic-sparing effects when used in rabbits. The aims of this study were to investigate the thoraco-lumbar anatomy of the rabbits, particularly the quadratus lumborum (QL) muscle, to design an appropriate US-guided quadratus lumborum block (QLB) specific for rabbits, and to define the most adequate volume of injectate required to consistently cover the ventral branches of T11 to L3 without affecting the pelvic limb innervation (L4, L5 and L6). Sixteen adult rabbit cadavers were included in the study. After randomization, four different volumes of injectate (0.1 mL/kg, 0.2 mL/kg, 0.3 mL/kg and 0.4 mL/kg) were tested, with these volumes additionally randomized to two sites of injection (right or left QL fascia). An ultrasound-guided QLB was performed with a solution of lidocaine, iodinated contrast and tissue dye (in a proportion of 3:1:1 volume, respectively), with subsequent computed tomography (CT) and anatomical dissection, to evaluate the spread of the injectate. In all but one case, the US-guided QLB performed with a dorsolateral approach using 0.3 mL/kg was adequate, while a dose of 0.4 mL/kg consistently reached the targeted nerves but also extended to L4 and caudally. This may suggest that an injectate volume of 0.3 mL/kg may be the most appropriate to produce adequate spread while not affecting pelvic limb innervation.

## 1. Introduction

Rabbits (*Oryctolagus cuniculus*) are the third most common pet in the UK, with an estimated population of 1.5 million [1]. It is therefore not surprising that there is a high demand to provide anesthesia and analgesia for surgical interventions and other invasive procedures in this species [2]. Despite this popularity, the anesthesia-related mortality in rabbits is still higher (1.39–4.8%) than in dogs (0.17%) and cats (0.24%), mainly due to a higher risk of gastrointestinal and respiratory complications reported in rabbits [3,4].

Rabbits are prey animals that tend to hide signs of disease and/or pain, which makes their clinical evaluation before and after a surgical intervention particularly challenging. The frequently misdiagnosed and/or undertreated pain in rabbits, in fact, may represent a contributing factor in the onset of significant physiological impairments that may compromise or delay the post-operative recovery in this species [5].

There is no sole technique to reduce morbidity or mortality as such; however, a multimodal analgesic approach has been shown to be effective in treating pain in dogs and cats, while reducing the amount of systemic analgesic drugs required and their possible side-effects [6]. This reduction may be particularly important in rabbits given the potential detrimental impact of opioids on gastrointestinal and pulmonary function.

The use of ultrasound-guided interfascial plane loco-regional anesthesia has recently grown in veterinary medicine because it is relatively easy to perform and carries a low rate of complications [7,8,9]. The common aim of these preceding studies was to obtain adequate perioperative analgesia that would allow a significant sparing effect on anesthetic requirements during surgical intervention, and better preservation of cardiovascular and respiratory function, while significantly reducing the requirements for systemic analgesic drug administration, based on dosage and number of innervations needed [6].

In rabbits, in order to provide analgesia for the abdominal wall, the transversus abdominal plane block has been described in a cadaveric study [10]; however, this block does not provide visceral analgesia, as previously described in human medicine [11].

The ultrasound-guided (US-guided) quadratus lumborum block (QLB) is an interfascial plane block developed to promote abdominal wall and visceral analgesia. The QLB consists of an injection of local anesthetic within the quadratus lumborum fascia, between the quadratus lumborum (QL) and the psoas major muscle (PM), with the aim to desensitize the ventral roots of the thoraco-lumbar spinal nerves and the sympathetic trunk to produce somatic and visceral analgesia of the abdomen. The QLB has gained a high acceptance in humans [12] and in veterinary medicine, where the QLB technique has been designed for different species: goats [13], minipigs [14], dogs [15] and cats [16]. Multiple approaches to reach the same targeted interfascial plane have been described in veterinary species: the transverse approach [17], longitudinal approach [18], dorsal approach [19] and, recently, with the needle passing through the erector spinae muscles [20]. 

While anatomical studies in dogs and cats are available, no study has investigated the anatomy of the thoraco-lumbar area and, particularly, the quadratus lumborum muscle and fascia in rabbits, which is an essential step in order to use the QLB technique safely in clinical cases.

According to the literature, the QL muscle in dogs is located directly ventral to the bodies of the thoracic vertebrae (T) T11, T12 and T13, and ventral to all of the lumbar vertebrae (L) [21]. The limited literature regarding the QL muscle in rabbits [22] reports that it originates from the bodies of the last four thoracic vertebrae (T9-T12) and terminates with the insertion in the transverse processes of the L6 vertebrae and the ventral angle of the iliac wing [22]. Species-specific anatomical differences and possible complications should be carefully evaluated before applying a QLB technique developed for other species such as humans or canines in rabbits.

In fact, despite the positive outcomes reported in the literature, the ultrasound-guided QLB has been associated with the desensitization of the femoral nerve in humans with temporary hindlimb motor function impairment [23]. In rabbits, the pelvic-limb motor nerve blockade may drive stress due to the inability to express normal behaviors, which may delay the recovery period and increase the post-operative complications [24].

In rabbits (as in dogs, cats and humans), the sciatic and the femoral nerves contribute to pelvic-limb motor function [25,26,27]. In rabbits, these nerves arise from the lumbosacral plexus, with the femoral nerve originating from the ventral branches of L4-L5-L6 and the sciatic nerve from the ventral branches of L6-L7 and sacral vertebra (S)S1-S2-S3 [26,27,28]. It is desirable that a QLB technique specifically designed for rabbits spares hindlimb motor function while providing adequate abdominal wall and viscera analgesia.

The aims of this study were

(1) To investigate the thoraco-lumbar anatomy of rabbits, particularly the QL muscle and fascia.
(2) To design an appropriate US-guided needling technique specific for rabbits.
(3) To ascertain the volume of injectate required to consistently cover the ventral branches of T11 to L3 without affecting those nerve branches responsible for innervation of the pelvic limb.

For these reasons, a cadaveric study in rabbits was conducted using the following steps:

(1) Anatomical dissections focusing on the QL and psoas muscles, thoraco-lumbar fascia, quadratus lumborum fascia and ventral branches of the associated spinal nerves.
(2) Sono-anatomical study and design of an ultrasound-guided QLB approach appropriate for this species.
(3) Comparison of the spread obtained using four different volumes of injectate via anatomical dissections and computed tomography (CT) scans.

We hypothesize that the US-guided QLB performed with the approach designed, using one of the volumes of injectate under investigation, will adequately and consistently stain the ventral branches of the spinal nerves from T11 to L3 without staining L4-L5-L6-L7-S1-S2-S3 (origin of the femoral and sciatic nerves).

## 2. Materials and Methods

This study was performed in a UK-based Veterinary Referral Hospital (Southern Counties Veterinary Specialists) between November 2022 and January 2023. For the study purposes, intact adult rabbit carcasses were obtained from a commercial UK-based distributor of frozen food for falconer, zoo and exotic purposes (www.kiezebrink.co.uk (1 November 2022)). Sixteen adult rabbits (two rabbits for the initial anatomical study, four rabbits for the QLB technique and CT study design, 10 rabbits for the injectate volume comparison study), of varying body conditions, gender and breed, were obtained. No animals were euthanized for the purpose of this study and no ethical committee approval was needed, as this was only a cadaveric study.

The carcasses were thawed for 48 to 72 h in a controlled-temperature room with an intermittent check of the intramuscular temperature (Meat thermometer, Umedo 2, Cardiff, UK) (in the quadratus femoris, vastus lateralis). All carcasses’ intramuscular temperatures were at least 18 degrees Celsius before any procedure was performed. For the rabbits undergoing US-guided QLB, both the thorax and abdomen were clipped.

### 2.1. Gross Anatomical Investigation

The first two carcasses were used for gross anatomical dissection to evaluate the quadratus lumborum muscle localization, its insertion sites and its relationship with the surrounding muscles, vertebrae and ribs.

**Figure 1:**
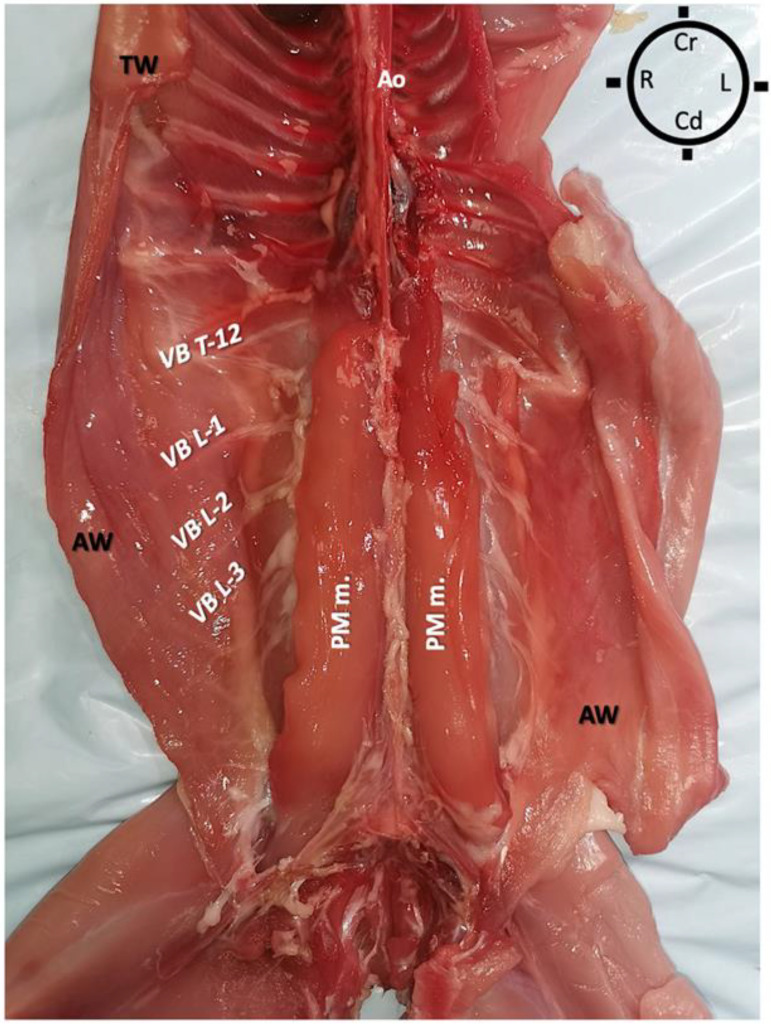
Skinned rabbit in dorsal recumbency showing both abdominal and thoracic viscera removed to expose the psoas compartment muscles. On the right and left sides of the dorsal midline, the psoas major muscle (PM m.) was identified originating from the last three thoracic vertebral bodies and respective ribs to insert on the femoral lesser trochanter (not shown). The psoas minor muscle was also identified (not shown). AO aorta; AW Abdominal wall; Cd Caudal; Cr Cranial; L Left; L- lumbar vertebrae; PM m. psoas major muscle; R Right; T- thoracic vertebrae; TW Thoracic wall; VB vertebral branches of spinal nerve.

The rabbits were placed in dorsal recumbency, and a ventral midline incision was performed to gain access to the peritoneal cavity. The abdominal viscera were all intact and were carefully removed to expose the retroperitoneal space and sub-lumbar muscles without damaging the psoas compartment muscles. To improve the visualization of the most cranial quadratus lumborum muscle insertion, both the diaphragm and ribs were dissected, and all thoracic viscera were also removed (Figure 1).

### 2.2. Preliminary Sono-Anatomy and Needling Approaches

**Figure 2:**
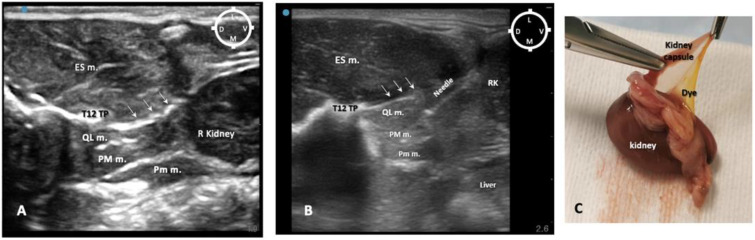
Ultrasound-guided quadratus lumborum block (QLB) performed using a ventromedial approach. (**A**) Sono-anatomy of the thoracolumbar area at the level of the twelfth thoracic vertebra transverse process, arrows pointing out the thoracolumbar fascia, hyperechoic line. (**B**) In a different rabbit cadaver, the QLB was performed, using the ventromedial approach injecting a mixture of lidocaine 2%, permanent yellow tissue dye and iodine contrast (3:1:1). The needle is visible on the ultrasound image travelling close to the right kidney (RK). (**C**) After anatomical dissection, in the same rabbit of (**B**), the right kidney was exposed and part of the mixture was found in the kidney capsule after injection. ES m. erector spinae muscles; D dorsal; L lateral; M medial; PM m. psoas major muscle; Pm m. psoas minor muscle; QL m. quadratus lumborum muscle; RK right kidney; T12 TP transverse process of the twelfth thoracic vertebra; V ventral. Cyan blue dot indicates the marker of the ultrasound probe pointed dorsally.

The first two out of four rabbit cadavers were used to understand the sono-anatomy and the relationship of the quadratus lumborum muscle and the thoraco-lumbar fascia surrounding the quadratus lumborum muscle and psoas muscles, and to design an ultrasound-guided quadratus lumborum muscle block approach specific for rabbits. For the needling, a ventral approach as reported in dogs and cats was first used [16,17] with the needle directed from the ventro-lateral to dorso-medial. However, during the sono-anatomy study, the abdominal structures such as the kidney and liver were very close to the needle path, resulting in an increased risk of potential internal organ damage (Figure 2A,B). Those concerns were confirmed during the anatomical dissections, as the dye solution was present within the kidney capsule (in three out of four thoraco-lumbar areas of two rabbits), likely due to its perforation when inserting the needle (Figure 2C).

**Figure 3:**
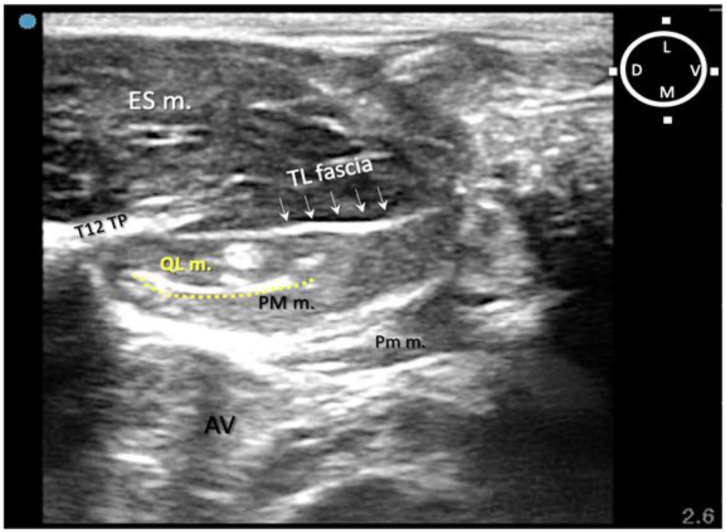
Sono-anatomy of the thoracolumbar area at the level of the twelfth thoracic vertebra transverse process (T12 TP). The arrows point out the thoraco-lumbar (TL) fascia (dot line). The quadratus lumborum muscle (QL m.) is located ventral to the transverse process and the TL fascia and dorsal to the psoas major muscle (PM m.). The injection point for the ultrasound-guided quadratus lumborum block is within the quadratus lumborum fascia the hyperechoic line separating the PM m. and the QL m (yellow dot line). AV abdominal viscera; D dorsal; ES m. erector spinae muscles; L lateral; M medial; Pm m. psoas minor muscle; V ventral; T12 TP Transverse process of the twelfth thoracic vertebra. Cyan blue dot represents the probe marker pointed in this case dorsally.

To overcome this complication, on the other two of four rabbits scheduled for the sono-anatomy and quadratus lumborum block design phase, the needling was performed using a modified dorsolateral approach as reported recently in dogs by Viscasillas and colleagues (2021), in which the needle passed through the epaxial muscles and a medial injection site was performed in the quadratus lumborum fascia, between the QL and PM muscles. This avoided visceral contamination with dye solution in these remaining two rabbits. For this reason, in the present study, only the dorsal approach was subsequently used (Figure 3).

### 2.3. Comparison of the Spread Using 4 Different Volumes

After the investigation of the QLB sono-anatomy, needling and approach, the ten remaining rabbits (20 hemi thoraco-lumbar areas, left and right) were randomized using a randomizer free-access online software (www.randomize.org (13 November 2022)) into four different volume groups (0.l mL/kg, 0.2 mL/kg, 0.3 mL/kg and 0.4 mL/kg). Those volumes were also randomized to the two sites of injection (right or left quadratus lumborum muscle) using a simple lottery system. These volumes were chosen based on previous studies performed in dogs and cats [18] with the aim to identify the lowest volume that would be sufficient to stain the ventral branches of the spinal nerves T11 to L3 while sparing those branches responsible for pelvic limb innervation.

Injections were performed ultrasound-guided using a veterinary-dedicated ultrasound system (S II Veterinary Ultrasound System, Fujifilm Sonosite, Bothel, WA, USA) using a linear probe (L25x, 13-6 MHz Linear transducer, Bothell, WA, USA) and a 21-gauge, 50 mm echogenic insulated peripheral nerve block needles (Echoplex, Vygon, Swindon, UK) attached to an extension line set, and a syringe containing the injectate mixture.

The injectate solution consisted of a mixture of lidocaine 2% (Lidocaine, Hameln Pharmaceuticals, Gloucester, UK), radiographic contrast medium (Iohexol 300 mg/mL, GE Healthcare Inc., Malborough, MA, USA) and tissue marking dye (yellow and orange, Mopec, Madison Heights, MI, USA) in a volume proportion of 3:1:1, respectively.

All ultrasound-guided QLBs were performed randomly by the same investigator, starting from the rabbit positioned in right lateral recumbency to perform the left QLB followed by left lateral recumbency to perform the right QLB. The ultrasound probe was placed transversely over the hypaxial musculature, caudal and parallel to the last rib, with the cranial marker pointed dorsally. The probe was then slid dorsally to visualize the transverse process of the L1 vertebra and its acoustic shadow, and this was used as a first reference point. As a second reference, the thoraco-lumbar fascia was identified and the aponeurosis followed up to the hyperechoic line between the QL and PM muscle, to identify the so-called quadratus lumborum fascia. Once the quadratus lumborum fascia (selected as the injection point) was identified, the needle was inserted using an in-plane technique with a dorsolateral to ventromedial direction, passing through the epaxial muscles and advancing the needle tip until it reached the thoraco-lumbar fascia first and then the quadratus lumborum fascia, between the PM and the QL muscles. To confirm the correct needle placement, a fraction of the dye solution (approximately 0.05 mL) was injected and, if hydrorelease of the tissues was observed, this was considered correctly positioned in the targeted area. If hydrorelease was not seen to separate the correct structures (QL from PM), the needle was repositioned backward or forward within the fascia until hydrorelease was achieved.

### 2.4. Computed Tomography Study

Within 10 min from the first ultrasound-guided QLB injection, each rabbit was positioned on the computed tomography (CT) table to perform the imaging studies to identify the injection site and the length of the contrast spread obtained. All CT studies were performed using a 64-slide Siemens SOMATOM Perspective (Siemens, Healthcare Limited, Erlangen, Germany).

To acquire the images, all rabbits were positioned in sternal recumbency with the pelvic and thoracic limbs extended; in all carcasses, the images obtained included the cranial thoracic spine to sacrum. The CT scan parameters included the following: helical acquisition; a slice thickness of 1 mm; a slice increment of 0.6 mm; a collimator pitch factor of 0.8 kVp140, 110 mA; a rotation time of 1.5 s; and a matrix size of 512 × 512. CT data were reconstructed to images with a 1 mm slice thickness using a medium-frequency (soft tissue (WW 350 HU, WL 40 HU)) and a high-frequency (bone window, window width (WW) 1500 HU, window level (WL) 300 HU) algorithm.

In the CT images obtained, the pattern of distribution of the contrast and the extension of distribution through the vertebrae were evaluated and recorded by the same investigators unaware of the volume used. All images were then interpreted by a European College of Veterinary Diagnostic Imaging board-certified clinician, who recorded the presence of the contrast in the interfascial plane between the quadratus lumborum and psoas muscles (yes/no), sympathetic trunk (based on vertebral level), in the epidural space (yes/no), and abdominal cavity (yes/no). All rabbits underwent anatomical dissection after CT.

### 2.5. Anatomical Dissection Following US-Guided QLB and Computed Tomography Study

**Figure 4:**
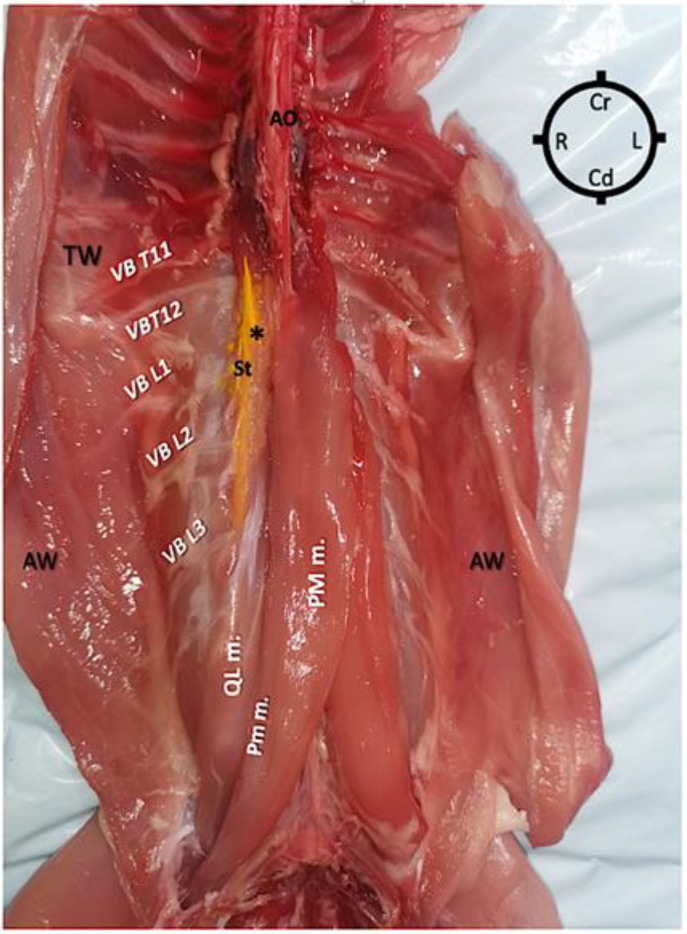
Ventral view of the dissected abdominal wall following removal of viscera of a rabbit carcass in which ultrasound-guided quadratus lumborum block was performed using a dorsolateral approach and a medial injection within the quadratus lumborum fascia. A volume of 0.3 mL/kg of injectate [lidocaine 2%, permanent yellow tissue dye, iodine contrast (3:1:1)] was injected into the quadratus lumborum fascia on the right side. The psoas major muscle (PM m.) has been bluntly dissected to expose the quadratus lumborum muscle (QL m.) and the injectate solution (*), which is distributed between the caudal eleventh thoracic vertebra and the third lumbar vertebra. It is possible to observe that the ventral branches (VB) of the spinal nerves from T12 to L3 were stained as well as the sympathetic trunk (St) partially visible in this dissection. AW abdominal wall; AO aorta; Cd caudal; Cr cranial; L left; R right; TW thoracic wall; VB T11, T12, L1, L2, L3: ventral branches of spinal nerves T-11, T-12, L-1, L-2 and L-3, respectively.

After obtaining the CT images, the carcasses were dissected to investigate the spread of the injectate mixture within the fascia. All dissections were performed by the same investigator, unaware of the injectate volume used. For the dissection, all rabbits were placed in dorsal recumbency and a ventral midline incision exposing the abdominal contents was performed. All abdominal viscera were carefully removed to expose the psoas compartment muscles. Blunt dissection of the PM muscle was performed to separate it from the QL muscle and expose the dye solution within the quadratus lumborum fascia. At this point, the spread of dye solution was evaluated by counting the number of stained ventral branches of spinal nerves and the correspondent number of vertebral bodies (Figure 4).

Staining of the nerves arising from T10, T11, T12, L1, L2, L3, L4, L5 and L6 were evaluated. The technique was deemed successful if all the ventral branches of the spinal nerves of T11 to L3 were stained and the dye solution was distributed around their circumference to a length greater than 0.6 cm [29], without further caudal dye spread, thus avoiding the nerves responsible for pelvic limb innervation.

In addition, complications such as intra-abdominal dye solution injection, spread to the spinal cord and injection in any of the surrounding organs were also recorded.

### 2.6. Statistical Analysis

The data were analyzed using the R-programming language (https://cran.r-project.org/bin/macosx/ (2 February 2023)). The parametric data were tested for normal distribution using the Shapiro–Wilk test and are presented as mean ± standard deviation (SD) and range. The Fisher’s exact test was performed to determine statistical significance using two categories: *category-1 adequate staining of the ventral nerve branches* (mix of lidocaine/dye/contrast staining all ventral branches of spinal nerves from T11 to L3, with L4 spared) and *category-2 inadequate staining of the ventral nerve branches* (extension of the staining cranial to T11 or T11 not reached, staining caudal to L3 or L3 not reached). 

A second Fisher’s exact test was performed to determine statistical significance using two other categories: c*ategory-1 adequate sympathetic block* (mix of lidocaine/dye/contrast staining cranial mesenteric ganglion and celiac ganglion plus the sympathetic trunk from T10 to L3) and *category-2 inadequate sympathetic block* (mix of lidocaine/dye/contrast not staining cranial mesenteric ganglion and/or celiac ganglion or not staining sympathetic trunk from T10 to L3). Adequacy was based on the veterinary literature [18] and with regard to the staining caudal to the L3 spinal ventral branches (L2-L3 intervertebral space); this was considered inadequate as it could affect the lumbar plexus, suppressing the rabbit’s motor function as reported in humans [23]. A *p* value < 0.05 was used to consider that the difference between groups was statistically significant.

## 3. Results

All cadavers were in satisfactory condition after thawing, and no significant damage was observed; therefore, they were all included in the study (16 carcasses in total, 10 of which were used for the volume comparison phase). The mean internal temperature of the cadavers was 20.0 degrees Celsius (°C) (range of 18 to 22 °C) and the mean carcass weight was 1.90 kg (range of 1.0 to 2.8 kg).

### 3.1. Gross Anatomical Investigation

**Figure 5:**
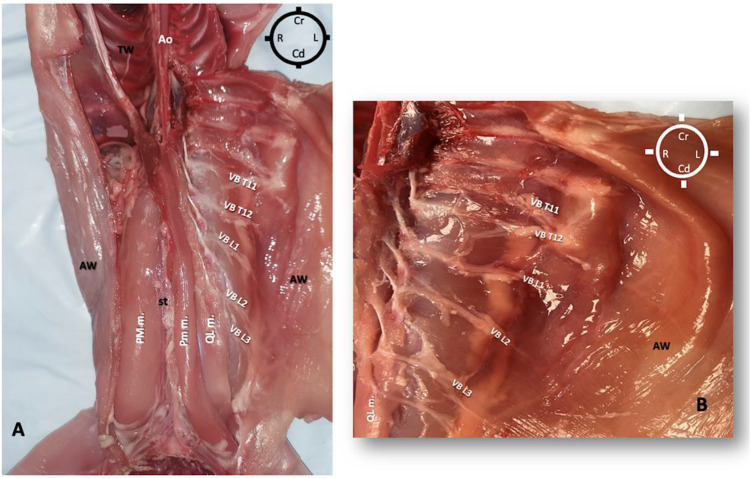
(**A**) Dissection of the left psoas major muscle (PM m.) revealing the quadratus lumborum (QL m.) and the psoas minor (Pm m.) muscles, as well as the ventral branches (VB) of the spinal nerves running into the transversus abdominis plane to innervate the abdominal wall (AW). (**B**) Details of the abdominal wall highlighting the ventral branches of T11, T12, L1, L2 and L3 running into the transversus abdominal plane (between transversus abdominis muscle and internal oblique muscle. Ao aorta; Cr Cranial; Cd Caudal; L Left; R Right. TW Thoracic wall; Ventral Branches of spinal nerves T11, T12, L1, L2 and L3, VB T11, 12, L1, L2, L3.

Dissection was performed by separating the PM muscle from the quadratus lumborum muscle (Figure 5A). In all cadavers, the QL muscles were intact and relatively easy to expose. The QL muscle was then followed from its cranial to its caudal bone insertions and the ventral branches of the spinal nerves were visualized running into the transversus abdominal plane (between the transversus abdominis and internal oblique muscle) (Figure 5A,B).

The QL muscle was localized dorsally to the psoas major muscle, laterally to the psoas minor muscle (Pm) and ventrally to the transverse processes of thoracic and lumbar vertebrae. It originated from the body of T7 and extended to insert in the transverse process of the lumbar vertebra L6 and the ventral angle of the iliac wing. The ventral branches of the spinal nerves were located and recorded as emerging from the intervertebral foraminae and running within a fascial plane surrounding the quadratus lumborum muscle between the QL and the PM muscles. Despite careful removal of the abdominal and thoracic viscera, the sympathetic trunk was not always left intact for its entire pathway in all cadavers, due to its very fragile nature. 

Using the computed tomography study, the spread of the contrast and the consequent staining of the sympathetic trunk were assessed.

### 3.2. Sono-Anatomy Study and US-Guided QLB Block Design

Once the approach and needling technique were designed, the ultrasound-guided QLB was performed at the first attempt within approximately 5 min per side in all 10 rabbits (20 sides). The ultrasound probe was initially positioned at the level of the thoraco-lumbar area (T12-L1) as reported in the literature [17], although due to the shape of the rabbits’ thoracic transverse processes (caudally, laterally and ventrally oriented), it was difficult to maintain a consistent probe position.

In order to overcome this issue, the probe was slid caudally toward the level of L1. Despite this adjustment, in some rabbits (2/10), the shadow of the T12 transverse process was still interfering with the sonographic image (upon anatomical dissection, the transverse processes of T12 in these two rabbits were considerably larger than the others). In these two rabbits, the ultrasound-shadow produced by the T12 transverse process at the level of T12-L1 space did not allow the visualization of the entire shaft of the needle during the approach; however, the tip was always visible. 

**Figure 6:**
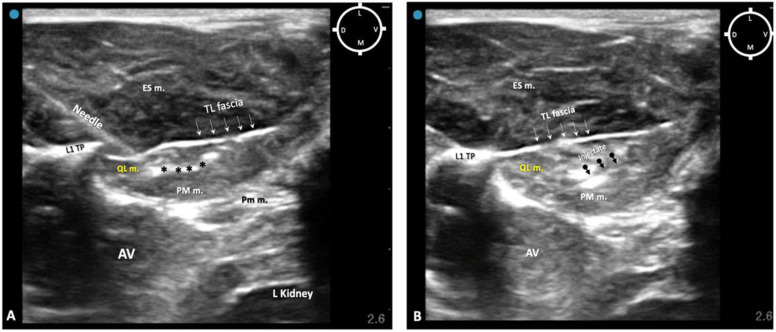
(**A**) Ultrasonographic image of the thoracolumbar area at the level of L1. The quadratus lumborum muscle (QL m.) is located ventrally to first lumbar vertebrae transverse process (L1 TP) and dorsally to the psoas major muscle PM m. The tip of the needle was introduced with a dorsomedial approach trough the thoracolumbar (TL) fascia aiming the quadratus lumborum fascia (asterisks) which separates the QL m. from the PM m. (**B**) Ultrasonographic image following injection of lidocaine 2%, permanent yellow tissue dye and iodine contrast (3:1:1) (black arrows) within the quadratus lumborum fascia separating PM m. from QL m. AV abdominal viscera; ES m. erector spinae muscles; D dorsal; L lateral; L Kidney left kidney; M medial; Pm m. psoas minor muscle; V ventral. Cyan blue dot represents the probe marker pointed dorsally.

In relation to the surrounding structures, the erector spinae muscles (particularly developed in rabbits) were located dorsally to the thoraco-lumbar fascia and the quadratus lumborum muscle, which was located dorsally and medially in relation to the psoas major muscle within the same acoustic window (range of depths between 1 and 2 cm). During the insertion of the needle, the tip and shaft were always visible in 8/10 rabbits (16/20 QLBs). In order to reach the targeted fascia, the needle was passed through the erector spinae muscles, pierced the thoraco-lumbar fascia, passed through the QL muscle and finally reached the target, the quadratus lumborum fascia. This fascia surrounds the QL muscle and separates it from the PM muscle. The mean depth of the targeted area of injection (QL fascia) was recorded as 1.5 ± 0.09 cm amongst all cadavers (Figure 6A,B).

### 3.3. Computed Tomography Study

The CT study revealed that in all cadavers at both sites, the contrast medium was detected in the interfascial plane between QL and PM muscles, surrounding the QL muscle in its ventral, lateral and medial aspects proportional to the volume of injectate used. The path of the injectate allowed in all the cases the separation of these two sub-lumbar muscles (QL muscle from PM muscle) in all rabbit cadavers. The dye distribution observed along the sympathetic trunk was statistically significant in the 0.3 mL/kg group compared with rabbits in the 0.1 mL/kg, 0.2 mL/kg and 0.4 mL/kg groups (Table 1).

Based on the CT images, the injection site occurred between T12 and L1 vertebrae, particularly at L1 (16/20), and at the level of T12-L1 (4/20).

According to the CT study, the distribution of the injectate was considered *adequate* when the injectate covered the cranial mesenteric ganglion, the celiac ganglion and sympathetic trunk from T10 to L3 vertebrae without further caudal or cranial distribution. The adequacy of the injectate spread to achieve a sympathetic trunk block evaluated by the CT study revealed that this was successfully achieved in 0/5 (0%) of the hemi-rabbits in the 0.1 mL/kg group; 0/5 (0%) of the hemi-rabbits in the 0.2 mL/kg group; 5/5 (100%) of the hemi-rabbits in the 0.3 mL/kg group; and 0/5 (0%) of the hemi-rabbits in the 0.4 mL/kg group. The spread of the contrast was not sufficient to cover the target area in rabbits in the 0.1 and 0.2 mL/kg groups, and a spread caudal to L3 occurred in rabbits in the 0.4 mL/kg group.

**Figure 7:**
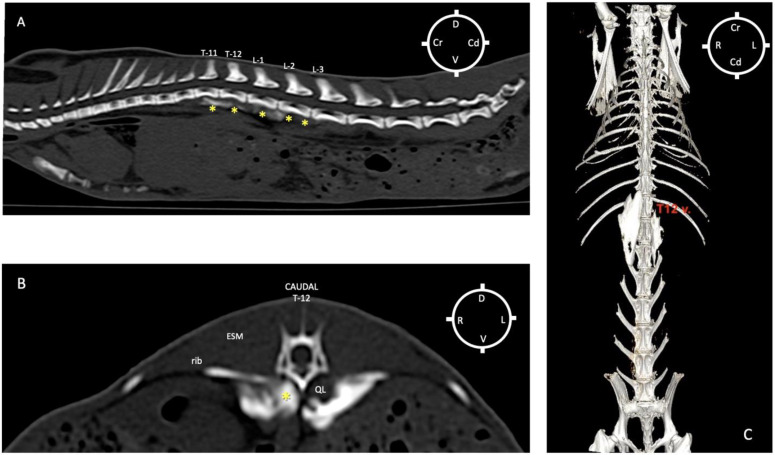
Computed tomography (CT) multiplanar study in a rabbit cadaver in which ultrasound-guided quadratus lumborum block was performed using a dorsal approach and medial injection of a volume of 0.2 mL/kg on the left side (L) and 0.3 mL/kg on the right side (R) of a mixture of lidocaine 2%, permanent yellow tissue dye, and iodine contrast (3:1:1). Injection point L1 bilaterally. (**A**) Sagittal multiplanar reconstruction image showing the distribution of the injectate (yellow asterisks) from eleventh thoracic vertebrae (T11) to intervertebral space between lumbar vertebrae L2-L3 (R side). (**B**) Transverse multiplanar reconstruction image showing the contrast distribution (yellow asterisk) at the level of caudal T-12 on both R and L sides with medial distribution, location of the sympathetic trunk, more pronounced in the R side comparing to the L side. (**C**) Three-dimensional reconstruction of the CT image of the same rabbit showing a linear pattern of distribution of the mixture on the R, and L hemi thoraco-lumbar area after QLBs. Cd caudal; Cr cranial; D dorsal; ESM erector spinae muscles; QL quadratus lumborum muscle; V ventral.

Overall, CT images showed that zero (0)/20 injections were in unwanted locations such as the abdominal cavities, surrounding organs, epidural canal or other muscle structures (Figure 7A–C).

### 3.4. Anatomical Dissection Following US-Guided QLB

In all cadavers, the injectate solution mix was injected between caudal T12 and L1 vertebral spaces, with the majority performed at L1 (16/20) (80%).

Based on the anatomical dissections of the carcasses, the aimed spread of the injectate (from T11 to L3 ventral spinal branches) was only obtained using 0.3 and/or 0.4 mL/kg of injectate. However, in 100% of the hemi-rabbits where 0.4 mL/kg was administered, the spread of the injectate also stained L4 ventral spinal branches and, in 60% of these carcasses, a caudal L4 spread, up to L6, was also found.

In regard to the 0.3 mL/kg group, 80% of the hemi-rabbits had a spread of the injectate at the level of cranial L3 vertebrae (4/5) and one at the L2-L3 intervertebral space area.

Regarding the lower volume of injectate groups (0.1 mL/kg and 0.2 mL/kg), the spread was inadequate: in the 0.2 mL/kg group, the spread of injectate missed the T12 ventral spinal branch in 20% of the hemi-rabbits, while in the 0.1 mL/kg group, the spread of injectate missed the T12 ventral branch in 40% of the hemi-rabbits, while the L3 ventral branches was never stained.

In terms of adequacy, the majority of rabbits (4/5, 80%) had the ventral branches of the spinal nerves from T11 to L3 stained with the injectate in rabbits in the 0.3 mL/kg group, whereas the results for the other volumes were more variable (Table 2).

The adequacy of spread of the dye/lidocaine/contrast mixture in rabbits in the 0.3 mL/kg group was statistically significant compared with rabbits in the 0.1 mL/kg, 0.2 mL/kg and 0.4 mL/kg groups (Table 2).

## 4. Discussion

This is the first anatomical ex vivo study investigating the anatomy and sono-anatomy of the quadratus lumborum muscle layout in rabbits and the first study describing an ultrasound-guided QLB for this species.

The findings of the present study showed that the QLB performed using a dorsolateral approach in rabbits was feasible and appeared safer compared to the ventrolateral approach described in other species [17], with the needle pathway sufficiently distant from vital organs such as the liver and kidney, which has the potential to provide a safe QLB in alive rabbits.

In regard to the adequacy of spread through the quadratus lumborum fascia obtained by injecting the mixture of local anesthetic, tissue dye and contrast while performing the QLB technique described here, the use of an injectate volume of 0.3 mL/kg resulted in staining of the ventral branches of the thoraco-lumbar spinal nerves from T11 to L3 in the majority of the cases (80%), as demonstrated by anatomical dissections.

In addition, CT images demonstrated that the sympathetic trunk and, in particular, the celiac ganglia were covered in 100% of the rabbits receiving 0.3 mL/kg of injectate. Furthermore, the volume of 0.3 mL/kg resulted in a sparing of the pelvic limb innervation in all cases.

The results of this ex vivo study produced a QLB approach specifically designed for rabbits that, using an adequate volume of local anesthetic, has the potential to provide visceral and abdominal wall analgesia without affecting the pelvic-limb motor function. The QLB designed here may potentially reduce the systemic opioid requirement and their attendant adverse effects in rabbits undergoing abdominal surgery; however, these findings need to be confirmed with a future in vivo study.

Besides the ability to desensitize the ventral roots of the thoraco-lumbar nerves, from T11 to L3, the ability to produce a sympathetic block is pivotal. In fact, although the true mechanism of the visceral analgesia provided by QLB has not yet been fully clarified, the current assumption is that the visceral analgesia results from the spread of anesthetics to the celiac ganglion/sympathetic trunk [30]. In the present study, the spread of the injectate was successful in staining the celiac and mesenteric ganglions, particularly when 0.3 mL/kg as the volume for the injectate was used.

In prey species such as rabbits, the visceral analgesia should be obtained, where possible, with a motor-sparing effect of the pelvic limbs. This is because the inability to move in conscious rabbits can be a cause of severe distress and even self-mutilation during the recovery period [32]. Future investigations are required to assess how the preservation of pelvic limb function following a QLB in rabbits potentially impacts their hospitalization time, levels of stress and post-operative food intake.

Regarding the anatomy of the quadratus lumborum region, in rabbits, the QL muscle runs from the body of T7 and extends to insert in the transverse processes of the lumbar vertebrae and to then insert on the ventral angle of the iliac wing, as has been previously reported [22]. This is similar to dogs, where the quadratus lumborum muscle is identified ventrally to the spinal column and extends through the bodies of the last three thoracic vertebrae (T11, T12, T13) and all the lumbar vertebrae until the iliac crest [17,20].

During the design of the needling technique, the authors began by using the ventrolateral approach, which was soon abandoned for the following reasons: 1. the transverse process of T12 vertebra in rabbits creates an intense ultrasound-acoustic shadow that does not allow the visualization of the entire shaft of the needle during advancement; 2. at this site, in order to be able to target the fascia, a degree of pressure needs to be applied with the probe to obtain a suitable image, which may cause ventilatory impairment in a live animal; and 3. the proximity of the abdominal viscera to the injection site [33] could potentially result in organ damage, as recorded in this study with penetration of the renal capsule. In fact, in human medicine, injury to the pleura, the kidney and retroperitoneal hematoma have all been reported with the ventrolateral approach [12,34].

For this reason, in all the subsequent QLBs performed in all other rabbits’ carcasses, a dorsolateral approach with middle-point injection between the QL and PM muscles within the quadratus lumborum fascia was used.

The dorsolateral approach resulted, in this study, in a feasible technique that allowed clear visualization of the shaft and tip of the needle, and no injection outside the targeted site was recorded on computed tomography or anatomical dissection. These results agree with the human literature where the incidence of complications is significantly lessened using the dorsolateral approach [25].

The QLB designed in this study showed a consistent spread of the injectate within the quadratus lumborum fascia in all cadavers, regardless of the volume of injectate used, as evaluated by computed tomography and subsequent anatomical dissection.

To perform the QLB reported here, the ultrasound probe was positioned at the level of the last thoracic vertebra as previously described [17], which, in rabbits, is T12 [22], as opposed to dogs, which is T13 [21]. Despite attempting to comply with current recommendations in the veterinary literature in terms of the level of the injection site for the QLB, these authors experienced difficulty in consistently achieving the same injection site in all subjects, and variability (between T12 and L1) was recorded in this study. The reason for this was potentially due to the anatomical variation between rabbits and dogs [21,22], with our dissections showing that rabbits’ transverse processes were oriented caudally, making it difficult to maintain the ultrasound needling plane with the ultrasound probe beam. In fact, in attempting to maintain the same injection site, the authors found it necessary to apply significant force with the probe against the T12 transverse process; however, the pressure required to maintain the probe in place while visualizing the targeted fascia would potentially compromise an alive patient’s ventilation. Therefore, the authors decided to perform this dorsolateral approach with middle-point injection into the QL fascia with the ultrasound probe slid more caudally using injection site L1 instead of between T12 and L1. This approach resulted in less acoustic shadowing from the T12 transverse process, and less pressure on the probe was required to visualize the relevant structures and the needle. Despite this adjustment, shadow interference from the T12 transverse process was still present in the ultrasound image in two out of ten rabbits. Despite species-specific ultrasound-guided loco-regional designed techniques, individual anatomic variability can still be present and further adjustments of the technique may be required.

Another interesting finding of the present study was the difference in terms of the injectate spread detected with CT images versus the anatomical dissection, with a positive correlation between the volume of injectate and the degree of discrepancy: for the lower volumes (0.1–0.2 mL/kg), the discrepancy between CT and anatomical dissection spread was negligible, while, with higher volumes (0.3–0.4 mL/kg), this was more relevant (±1 vertebral space). Similarly, a QLB study in dogs using a volume of 0.2 mL/kg [20] also reported a difference in detected injectate spread between the CT study and the anatomical dissections, with a more cranial distribution of the contrast during the CT study. These authors did not investigate this finding further; however, a possible explanation could be that while, during anatomical dissection, we were looking for the ventral roots of the nerves that, when exiting the vertebrae, are directed caudally, the CT enhanced the contrast present in the injectate mixture, resulting in a deeper and further spread within the structures despite its negligible volume.

These authors used the two techniques (CT and anatomical dissection) as each one provides different information: while the CT scan evaluates the spread of the injectate within the fascial planes, using the thoraco-lumbar vertebrae as a reference point and allowing sympathetic trunk and ganglions stain evaluation, the anatomical dissection, on the other hand, helps identify which ventral nerve branches are actually stained. The different reference point (ventral nerve branches vs. vertebral bodies) might have contributed to the discrepancy in the detected injectate spread between the two techniques in the present study.

The volumes of the dye solution used in this study were decided according to similar studies performed in dogs [15,16,17] and cats [16], but were, however, lower than some interfacial plane blocks [35] where a volume up to 0.6 mL/kg was used.

In the present study, a volume of 0.3 mL/kg resulted in an adequate spread, allowing consistent staining, in all but one case, of the ventral branches of the thoraco-lumbar spinal nerves from T11 to L3 without further spread to the L4 level; however, a volume of 0.4 mL/kg consistently stained L4 and more caudally. Moreover, a volume of 0.3 mL/kg consistently stained, in all cases, the sympathetic trunk and the cranial mesenteric and celiac ganglions. It is not possible to exclude that a volume between 0.3 mL/kg and 0.4 mL/kg would result in 100% of adequate staining of ventral nerve branches, and concurrently sparing the pelvic limb innervation, while producing an adequate sympathetic block. Further studies are needed in alive rabbits to determine the minimal volume and concentration of local anesthetic needed to obtain the desired analgesic effect without causing any side-effects due to local anesthetic absorption.

This ex vivo study has some limitations mainly related to the cadaveric nature of the investigation. Firstly, the population of rabbits used in this study was heterogeneous and not standardized for breed, size, gender or age, as they were obtained from a commercial distributor of frozen food for falconer, zoo and exotic animals. It is not possible to exclude that different results would be obtained if the same QLB is performed in a more homogeneous population such as New Zealand rabbits used for experimental studies.

There is also evidence that the distribution of the contrast and the local anesthetic may be different between cadavers and in vivo [15]. Freezing and thawing of cadavers may change the echogenicity of the fascial planes, muscles and vascular structures, which may not accurately reflect the in vivo sono-anatomy characteristics. In addition, an effect of temperature on the viscosity of the iodinated contrast has been reported [36]: the lower the contrast temperature, the higher its viscosity and the higher the injection pressure [36]. In the present study, all cadavers’ internal temperatures were between 18 °C and 22 °C, which may possibly contribute to a different injectate spread within the cadaver’s fascia when compared to live animals at their normal body temperature. The cadavers were only warmed to room temperature and not to their normal in vivo internal body temperature, to minimize cadaveric decomposition as much as possible, which could further affect the ultrasound images and anatomical dissection. Moreover, previous cadaveric studies used cadavers thawed at room temperature [16,17].

Lastly, the number of cadavers used in our study was relatively small and further investigations with larger samples are warranted.

## 5. Conclusions

The ultrasound-guided quadratus lumborum block in rabbits, performed with a dorsolateral approach and middle injection site within the quadratus lumborum and psoas major muscles as described in this study, resulted in adequate spread of the injectate within the quadratus lumborum fascia and staining of the ventral branches of the spinal nerves responsible for innervation of the abdominal wall and viscera. Furthermore, the staining of the sympathetic trunk and celiac and mesenteric ganglions obtained by performing the QLB described in the present study has the potential to produce an effective abdominal wall and visceral analgesia. Besides the analgesic effect that could be obtained by performing the QLB described here, using a dorsal approach, with 0.3 mL/kg as the injectate volume, in rabbits may spare their hindlimbs’ motor function in the post-operative period, allowing the expression of their natural behavior while reducing the risk of complications and the hospitalization.

Despite, among the volumes tested, the 0.3 mL/kg volume of injectate producing an adequate spread, in the majority of the cases (from T11 to L3, but sparing the hindlimbs’ innervation caudal to L3, as well as an adequate sympathetic trunk block), it is not possible to exclude that an injectate volume between 0.3 and 0.4 mL/kg would be able to produce adequate spread in 100% of the cases.

The QLB performed with the dorsolateral approach described here may also carry a lower risk of complications in live rabbits as the needling technique is performed away from major structures such as thoracic and abdominal organs. However, further in vivo studies are required to corroborate these results, and to evaluate if the use of this technique as part of a multimodal analgesia approach in rabbits undergoing abdominal surgery would facilitate post-operative recovery.

## Figures and Tables

**Table 1:** Distribution of the local anaesthetic/dye tissue/contrast mixture (3:1:1) pathway along the sympathetic nervous system evaluated with computer tomography, based on the vertebral level, in 20 hemi-rabbit cadavers where the QLB was performed using a dorsoventral approach and different volume of injectate (0.1 mL/kg, 0.2 mL/kg, 0.3 mL/kg and 0.4 mL/kg). The adequacy of the sympathetic block was based on the staining obtained. (Adequate equal to a staining of all the following structures; the cranial mesenteric ganglion; the celiac ganglion; and sympathetic trunk between T10 to L3). Data analysed with Fisher’s exact test, statistically difference between the groups detected; * *p*-value = 0.03.

Sympathetic Nervous System Structure (Vertebral Level)	0.1 mL/kg	0.2 mL/kg	0.3 mL/kg	0.4 mL/kg
Cranial mesenteric ganglion (L1-2 or L2-3) [30]	2/5	3/5	5/5	5/5
Celiac ganglion (T2-L1) [31]	3/5	5/5	5/5	5/5
Sympathetic trunk max spread	T12 to L1-2	T11 to L2-3	T10-11 to L3	T10 to L5-6
*Adequate* (Mesenteric and Celiac ganglions + sympathetic trunk from T10 to L3)	0/5	0/5	5/5 *	0/5

**Table 2:** Representation of the spread obtained after injection of four different volumes (0.1 mL/kg, 0.2 mL/kg, 0.3 mL/kg and 0.4 mL/kg) of injectate (mixture of lidocaine 2%, permanent yellow tissue dye, and iodine contrast (3:1:1)).Considered as adequate (spread covering the ventral spinal branches from T11 to L3) or inadequate (spread not covering the ventral branches of T11 or going more cranial than T11 or not reaching ventral branches of L3 or going more caudal than L3) based on the anatomical dissections. Data were analysed with Fisher’s exact test, statistically difference between the groups detected; * *p*-value = 0.017.

Spread	0.1 mL/kg	0.2 mL/kg	0.3 mL/kg	0.4 mL/kg
Adequate	0	2	4 *	0
Inadequate	5	3	1	5

## Data Availability

Data supporting those stated above can be sent to anyone requesting the authors.
